# Adolescent wine consumption is inversely associated with long-term weight gain: results from follow-up of 20 or 22 years

**DOI:** 10.1186/s12937-019-0478-7

**Published:** 2019-09-10

**Authors:** Pratiksha Poudel, Kamila Ismailova, Lars Bo Andersen, Sofus C. Larsen, Berit L. Heitmann

**Affiliations:** 1 Research Unit for Dietary Studies, The Parker Institute, Bispebjerg and Frederiksberg Hospital, The Capital Region, Copenhagen, Denmark; 2 School of Global Health, University of Copenhagen, Copenhagen, Denmark; 3 Department of Education, Arts and Sport, Western Norway University of Applied Sciences, Campus Sogndal, Bergen, Norway; 4 Department of Public Health, Section for General Medicine, University of Copenhagen, Copenhagen, Denmark; 5 The Boden Institute of Obesity, Nutrition and Eating disorder, University of Sydney, Sydney, Australia; 6 National Institute of Public Health, University of Southern Denmark, Odense, Denmark

**Keywords:** Alcohol, Adolescence, Obesity, Body mass index, And weight gain

## Abstract

**Background:**

Several studies have suggested a link between the type of alcoholic beverage consumption and body weight. However, results from longitudinal studies have been inconsistent, and the association between adolescent alcohol consumption long-term weight gain has generally not been examined.

**Methods:**

The study was based on data from 720 Danish adolescents aged between 15 to 19 years at baseline from the Danish Youth and Sports Study (YSS). Self-reported alcohol use, height, weight, smoking, social economic status (SES) and physical activity levels were assessed in baseline surveys conducted in 1983 and 1985, and in the follow up survey which was conducted in 2005. Multiple linear regression analyses were used to examine the association between alcohol consumption in adolescence and subsequent weight gain later in midlife.

**Results:**

There was no significant association between total alcohol consumption during adolescence and change in BMI into midlife *(P* = 0.079) (β − 0.14; 95% CI -0.28, 0.005). Wine consumption was found to be inversely associated to subsequent BMI gain *(P* = 0.001) (β − 0.46; 95% CI -0.82, − 0.09) while the results were not significant for beer and spirit. The relationship did not differ by gender, but smoking status was found to modify the relationship, and the inverse association between alcohol and BMI gain was seen only among non-smokers (*P* = 0.01) (β − 0.24; 95% CI -0.41, − 0.06) while no association was found among smokers. Neither adolescent nor attained socioeconomic status in adulthood modified the relationship between alcohol intake and subsequent BMI gain.

**Conclusion:**

Among non-smoking adolescents, consumption of alcohol, and in particular wine, seems to be associated with less weight gain until midlife.

**Trial registration:**

The YSS cohort was retrospectively registered on August 2017. (Study ID number: NCT03244150).

**Electronic supplementary material:**

The online version of this article (10.1186/s12937-019-0478-7) contains supplementary material, which is available to authorized users.

## Introduction

Adolescence is considered a sensitive period for future health as the brain still undergoes maturation [1]. Moreover, several behavioural traits including eating and drinking habits are formed during adolescence and are likely to sustain throughout life, which may be especially important in relation to adult risk of obesity [2].

Alcohol is considered to be a risk factor for obesity due to a high calorie content [3, 4], and because alcohol inhibits fat oxidation, which may result in accumulation of fat in adipose tissues [5]. On the other hand, alcohol is also known to have a high thermogenic effect that may result in increased energy expenditure [6]. Accordingly, results from previous studies examining the relationship between alcohol consumption and subsequent weight development are conflicting [7–15]. The discrepancy in results could partly be attributed to variation in types of alcohol beverage consumed. Studies have found mixed results for wine [16–19], and beer intake [20–22], while spirit intake was more consistently found to be directly related to risk of weight gain [16, 20, 23]. However, these studies were primarily based on adult populations and cannot be generalized to adolescents.

Wine is reported to be one of the most frequently consumed alcoholic beverages among adolescents [24, 25]. However, the evidence of a relationship between different types of alcohol during adolescent and body weight is limited. Most studies have examined total alcohol consumption, and either been cross sectional or lasted into young adulthood. Thus, it remains unclear whether adult obesity and weight gain into adulthood may be attributed to the types of alcoholic beverages consumed during adolescence. Of the few longitudinal studies conducted among adolescents, some found a direct association between high alcohol consumption and high self-reported weight gain [10, 11] while others found that adolescents with a high alcohol intake had a lower risk of becoming obese in young adulthood than adolescents with low intakes [12]. Most studies, however, did not account for type of alcohol consumed.

Thus, in the present study we examined the association between intake of total alcohol and type of alcoholic beverages (wine, beer, spirit) during adolescence and subsequent weight gain until midlife. We hypothesized that adolescent total alcohol consumption, and in particular beer and spirit consumption would be directly, and wine intake inversely, associated with weight gain into midlife.

## Materials and methods

### Study population

**Fig. 1:**
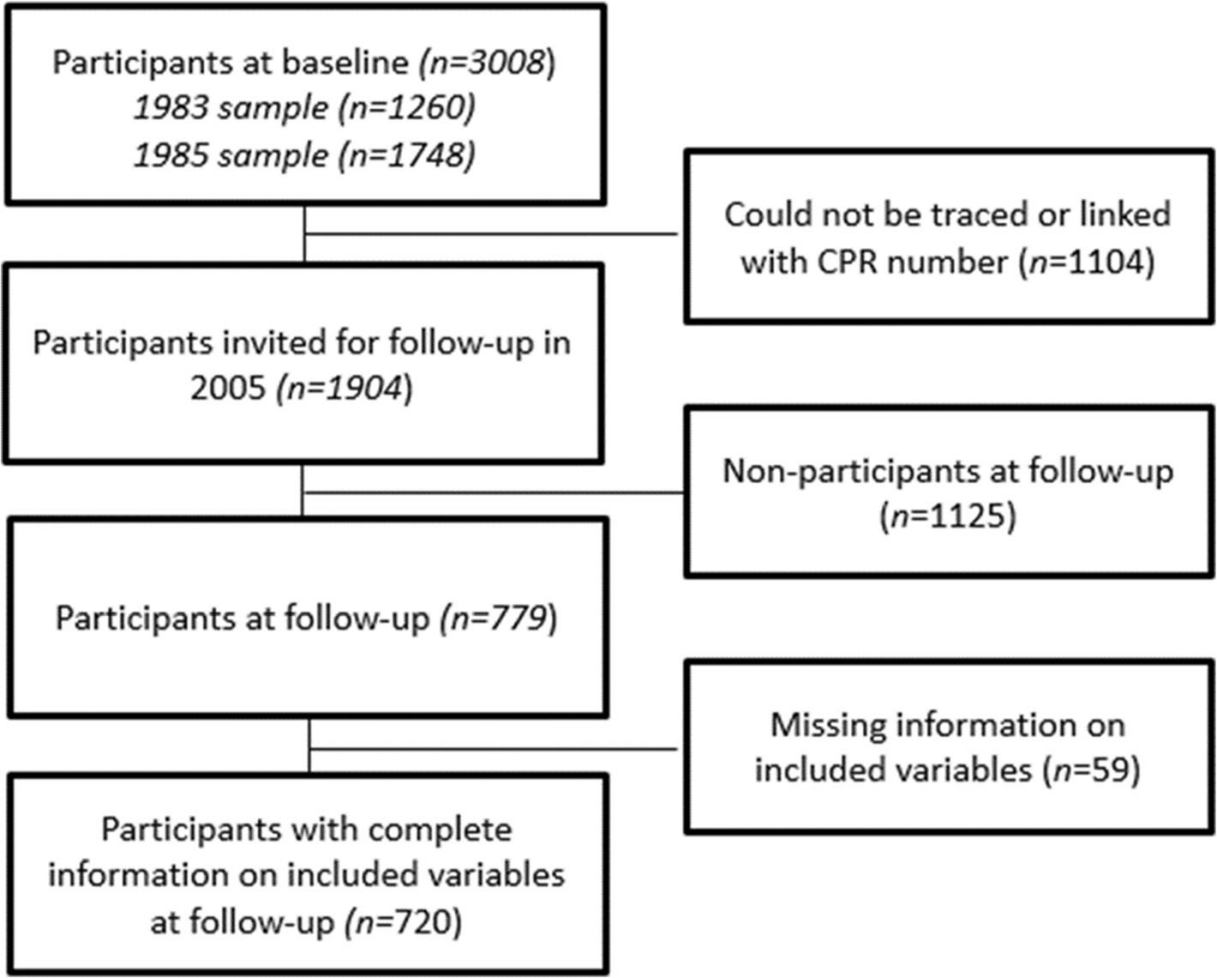
Participant flowchart from baseline to follow-up

The study was based on the Danish Youth and Sports Study (YSS), a 20 or 22 year follow up survey of 3008 Danish teenagers aged 15 to 19 and born between 1964 and 1969. Baseline data was gathered from students in high schools, vocational or trade school, who participated in surveys in either 1983 or 1985. Data on demographic, socioeconomic and lifestyle variables including self-reported height and weight, was collected through a set of questionnaires completed at the beginning of the study**.** Later, in 2005 a follow up survey was conducted, using similar set of questionnaires. A total of 1904 eligible participants were invited for the follow up survey of which 786 (41%) chose to participate. Data from the follow up survey was available for 779 participants. The remaining were either not traceable or chose not to participate. For the present study, participants were excluded if they had missing data on Body Mass Index (BMI) at baseline or follow up (*n* 35), had missing information on baseline socioeconomic status (SES) (*n* 21), or smoking status (*n* 1) and alcohol consumption (*n* 1). Moreover, one participant was excluded due to an extreme and unlikely baseline BMI value of 158. After exclusion of the participants with missing values on different variables, the final study population consisted of 720 participants (Fig. 1), of whom 294 were male and 426 were female.

### Measures

#### Alcohol consumption

The participants were asked to report their alcohol consumption by answering the question ‘How often do you drink different types (beer, wine, spirit) of alcohol?’. The questions were based on previously validated frequency questionnaire which sufficiently captured different types of alcohol consumption [26]. The participants responded by choosing one of the 7 categories (everyday, 2–3 times per week, once a week, twice per month, once a month, very rarely and never). The usage of three different types of alcohol (beer, wine, spirit) was then quantified with following assumptions: 1) Never- 0 standard drink per week; 2) Very rarely- 0.08 standard drink per week; 3) Once a month- 0.25 standard drink per week; 4) Twice per month- 0.5 standard drink per week; 5) Once a week- 1 standard drink per week; 6) 2–3 times per week- 2.5 standard drink per week; 7) everyday- 7 standard drinks per week. The reported alcohol usage was quantified as standard drinks and calculated as units (12 g alcohol) per week. All standard drinks were summed to create total alcohol consumption and used as continuous information in the analyses.

### Outcome assessment

#### Body mass index and obesity

BMI (kg/m^2^) was calculated from the self-reported height and weight in the baseline and follow-up surveys. Change in BMI was calculated as follow up BMI minus the baseline value and was included in the statistical analyses as continuous information.

### Covariate assessment

#### Smoking

The smoking status at baseline was assessed based on the question ‘Do you smoke?’ Smoking status was further classified as: current smokers, non-smokers and ex-smokers. The study participants were grouped into current smokers and non-smokers for the purpose of this study. Non-smokers included ex-smokers and never-smokers, and smokers included current smokers.

#### Socioeconomic status (SES)

Socio economic status at baseline was assessed based on questions on paternal occupation and education. The paternal SES was then assigned into five different categories according to the scale described by National Centre for Social Research (2016) [27]. 1) Persons with > 4 year of further education (master level), white collar workers with > 50 subordinates, and self-employed with > 20 employees; 2) Persons with short or medium further education (up to 4 years), white collar workers with 11–50 subordinates (if no master level education), and self- employed with 6–20 employees (if no master level education); 3) Persons with short education (up to 1 years), vocational education, white collar workers with 1–10 subordinates (if no education corresponding to SES group 1 or 2); 4) Skilled workers and white collar workers with no subordinates (if no education corresponding to SES group 1, 2, or 3); 5) semi-skilled and unskilled workers. We merged the SES groups into three groups. The first two highest SES groups were merged and named ‘high SES’, SES group 3 was named ‘middle SES’ and the two lowest groups were merged and named ‘low SES’. If no SES were available for father, the maternal SES was used (*n* = 26). Follow-up SES was based on participant’s own education and occupation.

#### Physical activity

Physical activity level was assessed at both the baseline and the follow up survey. The subjects were asked to report different types of sports and the total time spent per week on each sport. Leisure time physical activity was assessed based on the reported sports clubs they attended and the time they actively spent in each sport per week. Finally, the type and amount of time spent on activities other than school and sports club were also assessed. Each activity and sport were assigned a metabolic equivalent (MET) score, the ratio of work metabolic rate to a standard resting metabolic rate [28]. Based on MET score, the total energy expenditure from all activities relative to basal metabolic rate was calculated.

### Statistical analyses

Descriptive statistics was computed to describe sample characteristics of the study sample. Differences in participant characteristics according to gender were assessed using independent t-test for continuous variable and Chi-square test for categorical variables.

Multiple linear regression analyses were used to examine the association between total alcohol consumption or specific alcohol beverage consumption (independent variable) and midlife attained BMI (dependent variable). Data was tested for normality prior to performing the regression analysis. Model assumptions (investigating linearity of effects on outcomes, consistency with a normal distribution and variance homogeneity) were assessed for the fully adjusted models through residual plots. All analyses were performed in a crude and an adjusted model, covariates included adolescent SES, sex, physical activity (MET score), smoking status and baseline BMI. Covariates were chosen as a priori through potential association with predictors and outcome. Multicollinearity was assessed by variance inflation factor (VIF) before inclusion of the independent variables in the model which did not detect correlation among the variables (Additional file 1: Table S2). To examine the association between each beverage type and change in BMI, additional adjustment was performed for the types of alcoholic beverages by including all three types of beverages in the same model. Further, an interaction product term by sex, smoking, follow up alcohol consumption and baseline SES was added to the same model to examine possible interaction between the variables and alcohol consumption in relation to development in BMI. Stratified analyses were done if interaction effect was observed.

Results were considered statistically significant for *P* < 0.05 in the primary analyses. However, for interaction analyses *P* < 0.1 was used as the threshold for conducting stratified analyses, as the power to detect interaction effects was small due to the limited sample size.

All analyses were performed using statistical package for social sciences (SPSS v 22.0).

## Results

### Sample characteristics

The final sample consisted of 720 participants after exclusion of misreporting and incomplete data. Comparison of baseline characterises between excluded and included participants revealed no significant differences between the two groups (Additional file 1: Table S1).

Among the 720 participants, 41% were men and 59% were women (Table 1). At baseline, the mean (SD) age of male participants was 16.9 years (± 0.98) and female was 17.0 years (± 1.0). From baseline to follow-up, mean BMI increased by 4.8 kg/m^2^ and 3.5 kg/m^2^ in men and women, respectively, and similarly, total baseline alcohol consumption was higher in men than women, while baseline smoking was more frequent in women than men. No significant sex differences were found for physical activity level or SES of father.

**Table 1:** Participant characteristics stratified by gender

Characteristics	Male (40.9%)^a^ *n* = 294	Female (59.1%)^a^ *n* = 426	*P*-value^b^
Age (years)	16.9 (0.98)	17.0 (1.01)	0.23
BMI kg/m2 (baseline)	20.5 (2.1)	20.2 (2.2)	0.06
BMI kg/m2 (follow up)	25.3 (3.1)	23.7 (4.1)	< 0.001
Total alcohol intake (unit/wk)	2.06 (1.74)	1.56 (1.58)	0.04
Total beer intake (unit/wk)	1.02 (1.26)	0.63 (0.91)	< 0.001
Total wine intake (unit/wk)	0.70 (0.93)	0.65 (1.04)	0.001
Total spirit intake (unit/wk)	0.37 (0.45)	0.35 (0.46)	0.50
Physical activity level			
(MET scores)	4.73 (6.30)	5.34 (7.15)	0.25
SES (%)			0.10
Low	33.6%	41.1%	
Medium	29.5%	27.0%	
High	36.9%	31.9%	
Smoking (%)			0.03
Smoker	18%	25%	
Non-smoker	82%	75%	

^a^means ± standard deviation

^b^Independent t test for continuous variables and chi-square test for categorical variables

Table 2 shows the association between adolescent alcohol consumption and change in BMI into midlife. There was no significant association between alcohol consumption during adolescence and change in BMI into midlife (*P* = 0.079). The association remained essentially similar after adjustments for potential confounders (*P =* 0.058). Associations were similar for men and women (*P* for interaction = 0.79), for adolescent SES (*P for* interaction = 0.45), for adult SES (*P* for interaction = 0.56) and for alcohol consumption in midlife (*P* for interaction = 0.31*).* However, associations differed according to adolescent smoking status (*P* for interaction 0.09 without, and 0.10 with adjustment of the potential confounders). Accordingly, separate analyses for smokers and non-smokers were created and showed an inverse association between total alcohol consumption during adolescence and subsequent BMI change only among non-smokers both before (β − 0.22; 95% CI -0.39, − 0.04) and after (β − 0.24; 95% CI -0.41, − 0.06) adjustment for confounders, while associations among smokers were not significant (β 0.11; 95% CI -0.15, 0.36).

**Table 2:** Associations between adolescent alcohol consumption and subsequent change in BMI into midlife by smoking status in baseline and types of alcohol in crude and adjusted model

	*n*	Crude		Adjusted^b^	
		β (95% CI)^a^	*P*	β (95% CI)	*P*
Total alcohol intake	720	− 0.13 (− 0.27, 0.01)	0.079	− 0.14 (− 0.28, 0.005)	0.058
Smokers	157	0.05 (− 0.20, 0.30)	0.70	0.11 (− 0.15, 0.36)	0.41
Non-smokers	563	−0.22 (− 0.39, − 0.04)	0.01	− 0.24 (− 0.41, − 0.06)	0.008
Wine	660	− 0.52 (− 0.91, − 0.25)	0.001	− 0.46 (− 0.82, − 0.09)	0.01
Smokers	140	− 0.10 (− 0.74,0.53)	0.74	−0.20(− 0.96, 0.57)	0.61
Non-smokers	520	−0.79 (− 1.17, − 0.40)	< 0.001	−0.76 (− 1.19, − 0,33)	0.008
Spirit	667	− 0.21 (− 0.77, 0.36)	0.47	0.26 (− 0.38, 0.90)	0.43
Smokers	144	0.20 (−0.69, 1.09)	0.66	0.67 (−0.38, 1.72)	0.60
Non-smokers	523	−0.58 (− 1.334, 0.17)	0.13	− 0.13 (− 0.98, 0.69)	0.74
Beer	695	−0.02 (− 0.25, 0.22)	0.90	− 0.11 (− 0.36, 0.15)	0.41
Smokers	157	− 0.01 (− 0.42, 0.40)	0.95	−0.13 (− 0.57,0.31)	0.56
Non-smokers	538	−0.04 (− 0.32,0.25)	0.79	− 0.11 (− 0.42,0.20)	0.50

^a^The estimated coefficient is per unit increase in consumption of alcohol

^b^Adjusted for baseline SES, smoking (in combined analyses of smokers and non-smokers only), physical activity, baseline BMI, and sex. Analyses specific to alcohol types were additionally adjusted for other types of alcohol

Test results for association between the types of different beverage consumption and weight change into midlife are also presented in Table 2. An inverse association with subsequent change in BMI into adult life was observed for adolescent wine drinking (β − 0.46; 95% CI -0.82, − 0.09), while neither beer nor spirit intake was significantly associated with midlife BMI. Interaction test revealed significant differences in the association between wine and development in BMI for smokers and non-smokers (*P* = 0.06), with the strongest inverse association seen for the non-smoking adolescents before (β − 0.79; 95% CI -1.17, − 0.40) and (β − 0.76; 95% CI -1.19, − 0.33) after adjustment of potential confounders while for smoking adolescents an inverse but insignificant association was observed (β − 0.20; 95% CI -0.96, 0.57).

## Discussion

In this longitudinal analysis with 20 or 22 year follow up, we found that participants with higher wine consumption during adolescence subsequently gained less weight than those with a lower wine consumption. Furthermore, contrary to results from a previous study [11], in our study smoking status was found to modify the relationship between alcohol consumption and weight development where both total alcohol and wine drinking were associated with less gain in BMI into midlife for those who were non-smokers in adolescence, while no associations were observed for the smokers. The observed difference between smokers and non-smokers could have potentially been driven by differences in SES [29]. However, the association persisted after adjustment for SES and we found no evidence of an interaction between SES and alcohol intake, suggesting this was not the case in our study. Moreover, there was a considerably lower prevalence of smokers than non-smokers, and hence a lack of power may be responsible for the non-significant results among the smokers.

Results from previous prospective population studies looking at the association between adolescent drinking and subsequent weight gain are few and mixed, generally confined to weight development into young adulthood, only, or of cross-sectional nature [10–12, 15]. Hence, the present results are among the first to examine the long-term consequences of adolescent alcohol consumption and adult weight gain into midlife.

It has been suggested that alcohol consumption has short-term stimulatory effects on appetite and food intake, which may result in progressive weight gain [6]. Furthermore, it has also been demonstrated that alcohol temporarily inhibits fat oxidation resulting in accumulation of fat in the adipose tissue. These would promote an increased risk of developing obesity on the long run [30]. On the contrary, alcohol is also known to have a high thermogenic effect, which may result in increased energy expenditure following its intake [6]. It has also been suggested that some of the energy ingested as alcohol is ‘wasted’, due to the activation of the inefficient hepatic microsomal ethanol-oxidizing system [31]. These latter, might, in part, explain the observed inverse association in the present study. However, the inverse association observed could be also attributed to residual confounding, e.g. unrecorded differences in lifestyle and nutritional characteristics between those drinking more and less alcohol, wine in particular. Wine intake has repeatedly been associated with better overall nutrition and lifestyle, and moderate wine drinkers have been found to exhibit a better overall health and good quality of life [32]. On the other hand, others have found similar inverse associations between wine consumption and weight gain [16, 17, 20], suggesting that phytochemical compounds like resveratrol found in wine may offer additional protection against fat accumulation by inhibiting lipogenesis and de-regulating lipogenic gene expression [33, 34]. Moreover, resveratrol is known to increase insulin mediated glucose uptake thus, effectively helping to reduce blood glucose levels [34]. It is thus possible that the combination of antioxidants and ethanol in wine may have been responsible for the apparent benefits against weight gain from adolescence into midlife. This could also possibly explain why a similar association was not observed in relation to beer and spirit.

Strengths of the study include the detailed information on type of beverages and total alcohol consumption as well as the longitudinal nature and long follow up. However, the latter might also be a limitation as over such a long-time span, lifestyle and weight status may have changed several times during follow-up. Also, it can be argued that Danish adolescent’s lifestyle habits including their alcohol intake choices and patterns in 1983/85 were most likely different to today [35]. However, a potential biologic association between adolescent alcohol intake, which is occurring during a time in life that may leave imprinting of importance for long term weight development, would not be expected to be different. It is also a limitation that we did not have information on drinking patterns (e.g. binge drinking), as data was collected before it was apparent that not only quantity but also pattern may be of importance for health. Moreover, we used self-reported data and hence the study is potentially subjected to reporting bias. Previous studies have noted overestimation of self-reported height and under estimation of weight resulting under-estimated BMI [36] particularly among the overweight and obese [37]. The consequence of such a differential reporting bias among the overweight and obese is that significant associations may have been overlooked (as for total alcohol), and that “true” associations were even stronger than those observed (as for wine and weight gain). Although it has been noted that self-reported frequency of alcohol consumption seems adequately reliable and valid for the research purposes [38, 39] and compares well to peer based observation [39], information collected via diet surveys [40] or other questionnaires [40], both over and under reporting of alcohol in adolescence is still possible. This may have inflated our results and hence we could have seen significant associations that should have been insignificant (like for wine). However, whether this bias in reporting of alcohol was also related to adolescent BMI is unknown, and hence we cannot predict if reporting bias may have influenced our observed associations. Nonetheless, the results should be interpreted with caution as causation cannot be conferred due to the nature of study. Also, the varying size of glasses is unaccounted for in the study, which most likely may have attenuated the results.

Although we adjusted for several potential confounders, we can also not rule out that unmeasured or residual confounding remains. Though we would expect a high alcohol, but potentially not high wine intake, to be associated with poorer dietary and physical activity habits, we cannot exclude that an adolescent healthy lifestyle was associated with adolescent alcohol and wine intakes which may have been responsible for the long-term relationship with weight status into midlife.

Finally, response at follow up was 42% only, with the possibility of selection bias and thus the generalizability of these results is unknown. There was no differences in age between participants and non-participants (*P* = 0.25), while participants had slightly lower baseline BMI (20.6 vs. 20.3 kg/m^2^), and higher baseline SES (34 vs. 27%) [41]. Nevertheless, there is little reason to expect that the biological associations we observed in the present study between adolescent drinking and midlife cannot be generalised, as also suggested by the lack of modification or confounding by SES.

## Conclusion

Among non-smoking adolescents, alcohol, and in particular wine consumption, seems to be related to less weight gain until midlife. Whether this may be related to a specific lifestyle, including healthy diet and activity habits associated with wine and alcohol drinking in adolescence, or with functional properties in alcohol and wine cannot be concluded from the present study.

## Additional file

Additional file 1: **Table S1.** Baseline characteristics of excluded vs. included participants. **Table S2**. Variance Inflation factor (VIF) matrix for the independent variables. (DOCX 20 kb)

## Abbreviations

BMI: Body mass index
CI: Confidence interval
MET: Metabolic equivalent
SES: Socioeconomic status
YSS: Youth and Sport Study
