# Supplementary material for: Adolescent wine consumption is inversely associated with long-term weight gain: results from follow-up of 20 or 22 years
Source: Nutr J. 2019 Sep 10;18:56. doi: 10.1186/s12937-019-0478-7 (PMC6737643; doi:10.1186/s12937-019-0478-7)
Supplement: Supplementary file 1 — Table S1. Baseline characteristics of excluded vs. included participants. Table S2. Variance Inflation factor (VIF) matrix for the independent variables. (DOCX 20 kb) [file 12937_2019_478_MOESM1_ESM.docx]

Table S1 Baseline characteristics of participants in excluded vs. included participants

|  | Participants | | *P-value^b^* |
| --- | --- | --- | --- |
|  | Included (n=720) ^a^ | Excluded(n=59) ^a^ |  |
| Age (years) | 16.98(1.0) | 16.86(0.955) | 0.38 |
| BMI kg/m^2^(baseline) | 20.33(2.16) | 20.41(2.7) | 0.83 |
| BMI kg/m^2^(follow-up) | 24.36(3.78) | 24.80(4.49) | 0.45 |
| Physical Activity level (MET score) | 5.10(6.82) | 4.37(6.58) | 0.42 |
| Total alcohol | 1.61(1.75) | 1.33(1.24) | 0.36 |
| Beer | 0.80(1.08) | 0.67(0.78) | 0.39 |
| Spirit | 0.36(0.46) | 0.33(0.44) | 0.63 |
| Wine | 0.49(0.75) | 0.36(0.32) | 0.21 |
| SES% |  |  | 0.18 |
| Low | 38% | 23% |  |
| Medium | 28% | 37% |  |
| High | 34% | 40% |  |
| Smoking |  |  | 0.10 |
| Smokers | 22% | 31% |  |
| Non-smokers | 78% | 69% |  |

^a^ means ± standard deviation

^b^ Independent t test for continuous variables and chi-square test for categorical variables.

Table S2: Variance Inflation factor (VIF) matrix for the independent variables

|  | Sex | BMI (baseline) | SES (baseline) | Smoking | Physical activity level | Total alcohol |
| --- | --- | --- | --- | --- | --- | --- |
| Sex | - | 1.02 | 1.03 | 1.04 | 1.00 | 1.07 |
| BMI (baseline) |  | - | 1.03 | 1.05 | 1.01 | 1.07 |
| SES (baseline) |  |  | - | 1.05 | 1.01 | 1.05 |
| Smoking |  |  |  | - | 1.00 | 1.04 |
| Physical activity level |  |  |  |  | - | 1.08 |
| Total alcohol |  |  |  |  |  | - |
